# Gα_s_ signaling controls intramembranous ossification during cranial bone development by regulating both Hedgehog and Wnt/β-catenin signaling

**DOI:** 10.1038/s41413-018-0034-7

**Published:** 2018-11-20

**Authors:** Ruoshi Xu, Sanjoy Kumar Khan, Taifeng Zhou, Bo Gao, Yaxing Zhou, Xuedong Zhou, Yingzi Yang

**Affiliations:** 1 Department of Developmental Biology, Harvard School of Dental Medicine, 188 Longwood Avenue, Boston, MA USA; 2 State Key Laboratory of Oral Diseases, National Clinical Research Center for Oral Diseases, Department of Cariology and Endodontology, West China Hospital of Stomatology, Sichuan University, Chengdu, China; 3 Department of Orthopaedic Surgery, Guangdong Provincial Key Laboratory of Orthopedics and Traumatology, First Affiliated Hospital of Sun Yat-sen University, Guangzhou, China; 4 Department of Spine Surgery, Sun Yat-sen Memorial Hospital, Sun Yat-sen University, Guangzhou, China

## Abstract

How osteoblast cells are induced is a central question for understanding skeletal formation. Abnormal osteoblast differentiation leads to a broad range of devastating craniofacial diseases. Here we have investigated intramembranous ossification during cranial bone development in mouse models of skeletal genetic diseases that exhibit craniofacial bone defects. The *GNAS* gene encodes Gα_s_ that transduces GPCR signaling. *GNAS* activation or loss-of-function mutations in humans cause fibrous dysplasia (FD) or progressive osseous heteroplasia (POH) that shows craniofacial hyperostosis or craniosynostosis, respectively. We find here that, while Hh ligand-dependent Hh signaling is essential for endochondral ossification, it is dispensable for intramembranous ossification, where Gα_s_ regulates Hh signaling in a ligand-independent manner. We further show that Gα_s_ controls intramembranous ossification by regulating both Hh and Wnt/β-catenin signaling. In addition, Gα_s_ activation in the developing cranial bone leads to reduced ossification but increased cartilage presence due to reduced cartilage dissolution, not cell fate switch. Small molecule inhibitors of Hh and Wnt signaling can effectively ameliorate cranial bone phenotypes in mice caused by loss or gain of *Gnas* function mutations, respectively. Our work shows that studies of genetic diseases provide invaluable insights in both pathological bone defects and normal bone development, understanding both leads to better diagnosis and therapeutic treatment of bone diseases.

## Skull formation: Finding new avenues

Investigating two genetic diseases of skull formation revealed underlying similarities that may help improve treatments. During skull development, growth of too much or too little bone leads to deformation of the head or face, with potentially devastating medical consequences. To improve our understanding of the fine-scale processes that control bone deposition, Yingzi Yang at the Harvard School of Dental Medicine and co-workers investigated how bone formation occurs in progressive osseous heteroplasia (POH) and fibrous dysplasia (FD), which cause excessive and insufficient bone formation, respectively, during skull development. Using POH and FD model mice, they identified a single molecule underlying both diseases. Pinpointing this molecule allowed the researchers to identify drugs, previously developed for other diseases, that could help treat POH and FD; initial tests showed improved bone formation in both POH and FD model mice.

## Introduction

Identifying the cellular and molecular mechanisms whereby osteoblast cells are induced is centrally important in understanding the organizational principles underpinning a functional skeletal system. Deviation from the tight temporal and spatial regulation of osteoblast differentiation leads to a broad range of devastating diseases, such as craniosynostosis (premature suture fusion), heterotopic ossification (HO), and osteoporosis. In development, osteoblast differentiation is controlled by one of the two essential bone formation processes: intramembranous and endochondral ossification. During intramembranous ossification, mesenchymal progenitor cells differentiate directly into osteoblast cells, while during endochondral ossification, osteoblast differentiation is preceded by cartilage formation. The mechanisms underlying differential regulation of osteoblast differentiation in these two distinct ossification processes, although important, remain largely unknown. Molecular and cellular analyses of skeletal genetic diseases with abnormal osteoblast differentiation have provided important insights in the regulation of osteoblast induction, and in this study, we have focused on intramembranous ossification during craniofacial development. Progressive osseous heteroplasia (POH) (OMIM#166350) and Albright’s hereditary osteodystrophy (AHO, OMIM 103580) are caused by loss-of-function mutations in the *GNAS* gene, which encodes the stimulatory alpha subunit, Gα_s,_ heterotrimeric G protein that transduces signals from G protein-coupled receptors (GPCRs).^1,2^ POH and AHO are characterized by progressive extra-skeletal bone formation through an intramembranous process.^3,4^ In contrast, activating mutations of *GNAS* in McCune-Albright Syndrome (MAS) causes fibrous dysplasia (FD) (OMIM# 174800) characterized by reduced ossification and bone marrow fibrosis. Studies of both POH and FD have identified the novel roles of GPCR/Gα_s_ signaling in inhibiting Hedgehog (Hh) signaling while enhancing Wnt/β-catenin signaling in the regulation of osteoblast differentiation from mesenchymal progenitors.^1,2,5,6^ Activated Gα_s_ signaling has been found to reduce osteoblast maturation during endochondral bone formation,^2,5^ while loss of Gα_s_ signaling in committed osteoblasts resulted in severe osteoporosis characterized by impaired endochondral and intramembranous ossification due to accelerated differentiation of osteoblasts into osteocytes and decreased commitment of mesenchymal progenitors to the osteoblast lineage in association with attenuated Wnt signaling.^7^ In humans, Gα_s_ signaling likely plays important roles in normal craniofacial development as both AHO and FD patients exhibit severe cranial bone defects. In AHO patients, craniofacial malformation such as craniosynostosis has been observed^8^ and FD patients show craniofacial hyperostosis, which is characterized by polyostotic sclerosis and cystic changes in craniofacial bone.^9^ However, the cellular and molecular mechanisms underlying craniofacial bone defects in AHO or FD remained unknown. This is largely due to poor understanding of intramembranous ossification, which has hampered therapeutic development.

Calvarium development is tightly regulated at both molecular and cellular levels.^10–12^ Mammalian cranium, or neurocranium, is the upper and back part of the skull. It protects the brain and supports sensory organs such as the ear and viscerocranium that support the face. The neurocranium can be divided into calvarium and chondrocranium, which grows to be the cranial vault that surrounds the brain and the skull base, respectively. Calvarium is composed of flat bones: frontal bones, parietal bones, the inter-parietal, part of occipital bone, and squamous parts of temporal bone,^12,13^ all undergo intramembranous ossification. By cell lineage analysis in mice, frontal bones show major contribution from the neural crest (NC) cells, while parietal and inter-parietal bones originate from head mesoderm.^14–16^ Osteoblasts within calvarial bone primordia differentiate and secrete matrix, so that the calvarial bones grow and become mineralized^12^ and finally meet at suture lines or fontanels. Cranial malformations are often progressive and irreversible, many of which need aggressive surgical management to prevent or mitigate severe impairment such as misshapened head or abnormal brain growth.^10^ For instance, craniosynostosis (premature suture closure) is a significant medical problem and one of the most common cranial defects that affects 1 in 2 500 individuals and requires surgical correction. Identifying molecular pathways that control cranial bone formation and growth is critically important in targeted therapeutic development.^17^

As the ectopic bone in POH and AHO patients forms through intramembranous ossification, we hypothesized that the Gα_s_-Hh signaling axis identified in POH and AHO is a common pathway that critically regulates intramembranous ossification both in normal skull development and in HO patients. In addition, as Gα_s_ signaling also regulates Wnt/β-catenin signaling during osteoblast differentiation and maturation,^2,5^ we hypothesized that Gα_s_ signaling regulates intramembranous ossification by regulating both Hh and Wnt/β-catenin signaling. When Hh and Wnt/β-catenin signaling were manipulated in the mesenchymal progenitor cells or committed osteoblast cells, it has been found that while Hh signaling is required to induce osteoblast differentiation from osteoblast progenitor cells, Wnt/β-catenin signaling acts in the following steps to induce osteoblast commitment but inhibits further osteoblast maturation and ossification.^18,19^ Hh signaling is required for osteoblast differentiation,^20,21^ but its role in cranial bone development was not clear. Loss of Gα_s_ signaling induces osteoblast differentiation by activating Hh signaling during ectopic intramembranous bone formation under pathological conditions.^1^ However, loss of Indian Hedgehog (Ihh) ligand completely abolishes endochondral ossification but not intramembranous ossification during craniofacial bone formation.^20^ Furthermore, blocking ligand-dependent Hh signaling activity by removing a Hh receptor *Smoothened* (*Smo*) using the *Wnt1-Cre* mouse line resulted in severely reduced, but not completely abolished, formation of NC-derived craniofacial bone formation.^22^ Smo is required to transduce the signaling from all Hh ligands: Ihh, Sonic Hedgehog (Shh), and Desert Hedgehog (Dhh).^23^ Therefore, it appears to be differential regulation and/or requirement of Hh signaling during intramembranous ossification compared to endochondral ossification. We hypothesized that additional Hh-ligand-independent mechanisms may regulate craniofacial bone formation and Gα_s_ signaling is a strong candidate given the craniofacial malformation found in AHO and FD patients.

Here we have tested whether the mechanisms underlying pathological osteoblast induction by Gα_s_ signaling loss in genetic diseases may also be applied to normal intramembranous bone development. We found that Hh signaling was differentially regulated during cranial and long bone formation by Gα_s_ and Hh ligands. Both Hh and Wnt/β-catenin signaling mediated Gα_s_ signaling in cranial bone development and small molecule inhibitors of Hh and Wnt/β-catenin signaling rescued craniofacial phenotypes in POH and FD mouse models, respectively. Therefore, insights gained by studying abnormal bone formation in genetic diseases allowed us to identify novel cellular and molecular mechanisms underlying normal craniofacial bone formation and therapeutic targets to treat craniofacial birth defects.

## Results

### Reduced Gα_s_ signaling correlates with increased Hh signaling in the developing cranial bone

**Fig. 1:**
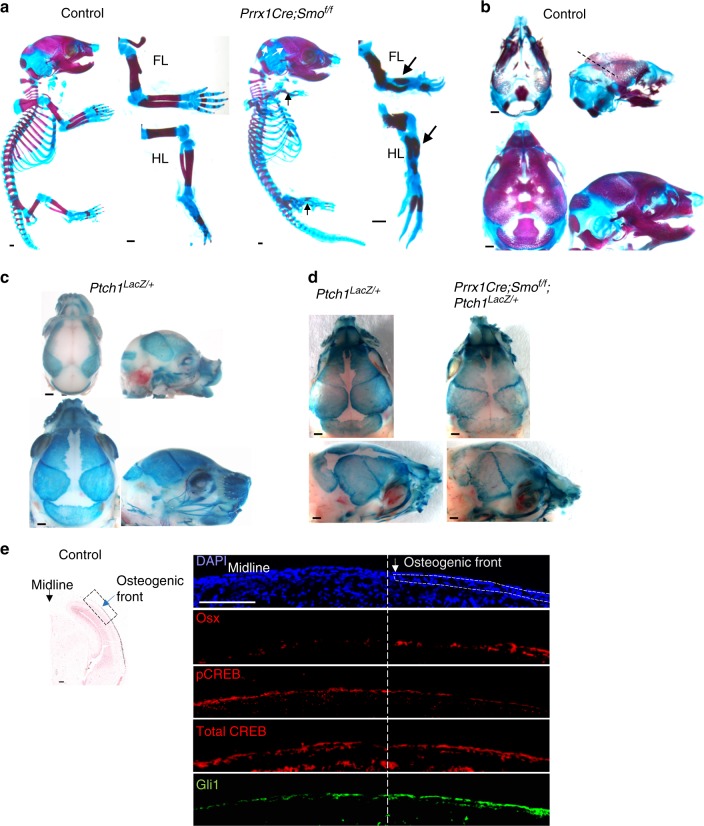
Hh signaling is activated in the forming calvarial bone in which Smo function is dispensable. **a** Alizarin red and alcian blue staining of controls and *Prrx1Cre; Smo*^*f/f*^ embryos at E18.5. The limbs are shown in high magnification on the side. Loss of long bone ossification in the limb (black arrow) and well-developed parietal bone and inter-parietal bone (white arrows) are indicated. **b** Alizarin red and alcian blue staining of control mouse heads from E15.5 and E18.5 embryos. E15.5 coronal sectioning positions (dotted lines) are indicated. Left panel: dorsal view; right panel: lateral view. **c** X-gal staining of mouse heads from the *Ptch1*^*LacZ/+*^ embryos at E15.5 and E18.5. Left panel: dorsal view; right panel: lateral view. Scale bar in **a**–**c**: 0.5 mm. **d** Whole-mount X-gal staining of mouse embryo head with the indicated genotypes at E18.5. X-gal staining was mildly reduced in mutants. Removal of *Smo* led to mildly reduced Hh signaling in the developing skull. **e** Coronal sections of the parietal bone at E15.5 (indicated in **b**) were stained with von Kossa method and Safranin O. The osteogenic front regions (dotted box) are shown in high magnification after being stained with the indicated antibodies. Leading edge of osteogenic front (white arrow and white dotted line) is indicated. Scale bar: 100 μm

To test the possibility that Hh signaling differentially regulates intramembranous and endochondral bone formation, we completely blocked ligand-dependent Hh signaling activity in both intramembranous and endochondral bone formation by removing *Smo* using the *Prrx1-Cre* mouse line. *Prrx1-Cre* targets the future posterior frontal bone, parietal, and inter-parietal bones that originate from the head mesoderm (Fig. S1a, b).^14–16,24^ In the *Prrx1Cre; Rosa 26*^*tdTomato*^ embryo at embryonic day 18.5 (E18.5; Fig. S1a), *Prrx1Cre*-targeted cells shown by tdTomato expression contributed to posterior frontal bones, parietal bones, inter-parietal bone, coronal suture, lambdoid suture, sagittal suture, anterior fontanel, and posterior fontanel at E18.5 (Fig S1a). Craniofacial elements derived from NC are shown in green, whereas the ones from mesodermal origin are shown in blue in a modified schematics according to a previous study^22^ (Fig.S1b). On this schematics, the Prrx1 lineage was indicated in red. These data indicate that the Prrx1 lineage are not only largely derived from mesodermal origin but also overlap with NC origin in the posterior frontal bone. In addition, the Prrx1 lineage cells include all Osx+ cells and other surrounding progenitor cells in the targeted region (Fig. S1c). We found that, in the *Prrx1-Cre; Smo*^*f/f*^ embryos, while endochondral bone formation in the limb was similarly blocked as in the *Ihh*^*−/−*^ embryos,^20^ cranial bone formation controlled by intramembranous ossification was mildly affected, with good morphology and mildly reduced sizes (Fig. 1a). These results indicate that ligand-dependent and ligand-independent Hh signaling pathways both regulate skull bone development and their relative contributions to the formation of skull bones from different origins may vary. We therefore examined Hh signaling activity in the developing cranial bone using a *LacZ* “knock in” allele of *Ptch1*.^19^ As *Ptch1* is a transcriptional target of Hh signaling,^25^ *LacZ* expression in the *Ptch1*^*LacZ/+*^ mice, which can be visualized by X-gal staining, serves as an in vivo readout of Hh signaling activity.^19^ X-gal staining of *Ptch1*^*LacZ/+*^ embryos at the E15.5 and E18.5 was performed. Analyses were focused on the frontal and parietal bones, in which ossification extended gradually from the basolateral region to the apex of the skull during development (Fig. 1b). Strong X-gal staining was first found in the basal–lateral region at E15.5, extended to the apex of the skull at E18.5 in the *Ptch1*^*LacZ/+*^ embryo (Fig. 1c). Interestingly, when Smo was removed by Prrx1-Cre, Hh signaling activities were still present, albeit weakly reduced when X-gal staining was performed in the E18.5 *Prrx1-Cre; Smo*^*f/f*^*; Ptch1*^*Lacz/+*^ and the control *Ptch1*^*Lacz/+*^ developing skulls (Fig. 1d). These results indicate that Hh signaling is strongly activated during intramembranous bone formation and suggest that Ihh- and Smo-independent activation of Hh signaling may control osteoblast differentiation during normal craniofacial bone development and growth.

Gα_s_ signaling has been shown to inhibit Hh signaling downstream of Smo by inhibiting Gli activities during ectopic bone formation in POH patients and in brain tumors.^1,26^ It is likely that the role of Gα_s_ signaling in ectopic bone formation reflects its underappreciated function in regulating normal intramembranous ossification through inhibiting Hh signaling, as both ectopic bone in POH and normal cranial bones undergo intramembranous ossification. To test this hypothesis, we examined Gα_s_ and Hh signaling at the osteogenic front of the developing parietal bone at E15.5 by immunofluorescence staining (Fig. 1e). Osteoblast differentiation was marked by an early osteogenic differentiation marker Osterix (Osx) expression,^27^ which was gradually increased from the osteogenic front to the mature bone, indicating a gradient of osteogenic differentiation. Gα_s_ signaling was shown by detecting CREB phosphorylation (pCREB), which is a readout of protein kinase A (PKA) activation by Gα_s_ signaling. Phosphorylated CREB (pCREB) was gradually reduced while the total CREB levels did not change, suggesting that Gα_s_ signaling was gradually decreased from the osteogenic front as the bone matured. Hh signaling was examined by the expression of Gli1, a readout of Hh signaling.^28^ Importantly, Gli1 expression was increased while pCREB levels were reduced from the osteogenic front to the mature bone. These results show that, while osteoblast induction is associated with Hh signaling activation, there is an inverse correlation between Gα_s_ and Hh signaling during cranial bone formation.

### Activation of Gα_s_ signaling inhibits cranial bone formation

**Fig. 2:**
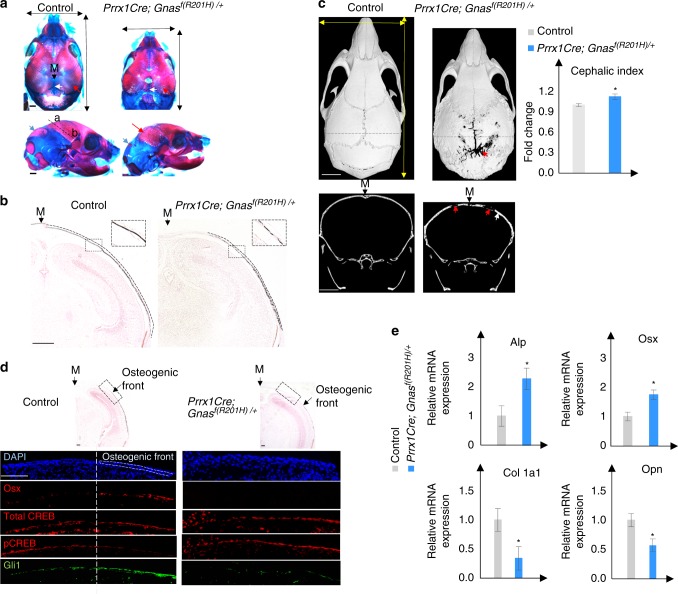
Gα_s_ activation delays intramembranous ossification. **a** Alizarin red and alcian blue staining of the mouse heads from embryos at E18.5. Increased suture space (white arrow) and reduced parietal and inter-parietal bone (red and blue arrow) are indicated. Scale bar 0.5 mm. Maximum length and width of the head (double arrows) are reduced in the mutant. a apex, b base. **b** von Kossa and Safranin O staining of parietal bone sections from E18.5 embryos; section position is indicated in **a** by a dotted line. M midline (black arrow). Mineralized parietal bone region is indicated by dotted lines. Boxed region is shown in higher magnification at the upright corner. Scale bar: 500 μm. **c** μCT image of 2-month-old mice. Upper panel: Dorsal view of a three-dimensional (3D) reconstructed image. Enlarged fontanel (red arrow) is indicated. Lower panel: Coronal two-dimensional (2D) image at a position indicated by the dotted lines in the upper panel. Thickened bone (white arrow) and bone gaps (red arrow) are indicated. Scale bar: 5 mm. Right panel: Cephalic index of the skull from 2-month-old mice. Cephalic index = 100 × width/length. Results are shown as average of measurements of three different mice±SD. *P < 0.05. **d** von Kossa and Safranin O staining of parietal bone from E15.5 embryos. The boxed osteogenic front region in the WT embryo and a region in the same position in the mutant were processed for immunofluorescent staining with the indicated antibodies. Images are shown in the lower panel. Osteogenic front is indicated (dotted area). Scale bar: 100 μm. **e** qRT-PCR analysis of osteoblast differentiation genes in the parietal bone tissues from P0 mice. Results are shown as average of three independent experiments±SD. *P < 0.05. The two-tailed Student’s *t* test was used in the statistic analysis

We next asked whether Gα_s_ signaling indeed plays an important role in cranial bone formation. We hypothesized that, Gα_s_ signaling activation by *Gnas* ^*R201H*^ expression^5^ in early osteochondral progenitor cells in mice should delay bone formation, while deletion of *Gnas* using a floxed loss-of-function *Gnas* allele (Gnas^f^)^29^ should accelerate bone formation during embryonic development of the cranial vault. To test our hypothesis, the *Gnas*^*f(R201H)/+*^ mice^5^ were crossed with the *Prrx1-Cre* mouse line.^24^ Cranial bone formation was examined at E18.5 by skeletal preparation and von Kossa staining of the cranial bone section (Fig. 2a, b). Compared to the littermate control of *Prrx1-Cre* or *Gnas*^*f(R201H)/+*^ embryos or mice, which are phenotypically indistinguishable from the wild-type ones in development, the *Prrx1-Cre; Gnas*^*f(R201H)/+*^ mutant embryos showed delayed ossification expansion from the base to the apex of the skull, therefore parietal or inter-parietal bone formation was reduced. Mineralization, assessed by von Kossa staining, was also reduced as indicated by the “sand patch” pattern in the *Prrx1-Cre; Gnas*^*f(R201H)/+*^ mutants (Fig. 2b). Reduced bone formation was also observed in the postnatal cranial bone at 2 months of age by micro-computed tomographic (μCT) scanning (Fig. 2c). Similar to what has been reported in CT scans of human polyostotic fibrous dysplasia,^30^ the *Prrx1-Cre; Gnas*^*f(R201H)/+*^ mutant skull showed mixed-density fibrous dysplasia lesions with mixed lucencies and sclerosis (arrows in Fig. 2c). Reduced bone formation was also manifested by reduced head length and width (Fig. 2a, c). The mutant phenotype was further quantified by the cephalic index (CI) or cranial index, which is the ratio of the maximum width (biparietal diameter, side to side) of the head multiplied by 100 divided by its maximum length (occipitofrontal diameter, front to back). The CI of the *Prrx1-Cre; Gnas*^*f(R201H)/+*^ mutant heads was increased comparing to the littermate control, suggesting that activated Gα_s_ signaling may have caused brachycephaly.^31^

To determine Gα_s_ signaling activity at the osteogenic front in the *Prrx1-Cre; Gnas*^*f(R201H)/+*^ mutants, we examined the levels of pCREB and total CREB at E15.5 (Fig. 2d). Indeed, in a location similar to the osteogenic front in the wild-type control, we found that, in mutants, while there was no change of the total CREB levels, pCREB levels were increased. In addition, Osx expression was not detected, while Gli1 expression was reduced in the mutant (Fig. 2d, S6b). These results indicate that *Gnas*^*R201H*^ expression resulted in increased PKA activity, reduced Hh signaling, and delayed osteoblast differentiation. Bone formation was further analyzed at P0 by quantitative real-time PCR (qRT-PCR) analysis of gene expression in the formed parietal bone as illustrated (Fig. S2a-c). Early osteoblast differentiation was examined by alkaline phosphatase (*Alp*) and *Osx* expression, which were both increased in the *Prrx1-Cre; Gnas*^*f(R201H)/+*^ mutants compared to the control (Fig. 2e). However, markers for mature osteoblasts such as *Collagen 1a1* (*Col1a1*) and *Osteopontin* (*Opn*) were reduced in expression in the *Prrx1-Cre; Gnas*^*f(R201H)/+*^ mutant (Fig. 2e). Therefore, Gα_s_ activation by *Gnas*^*R201H*^ expression in osteochondral progenitor cells during cranial bone formation inhibited Hh signaling, delayed osteoblast induction in early development, and blocked osteoblast maturation later in the formed cranial bone.

**Fig. 3:**
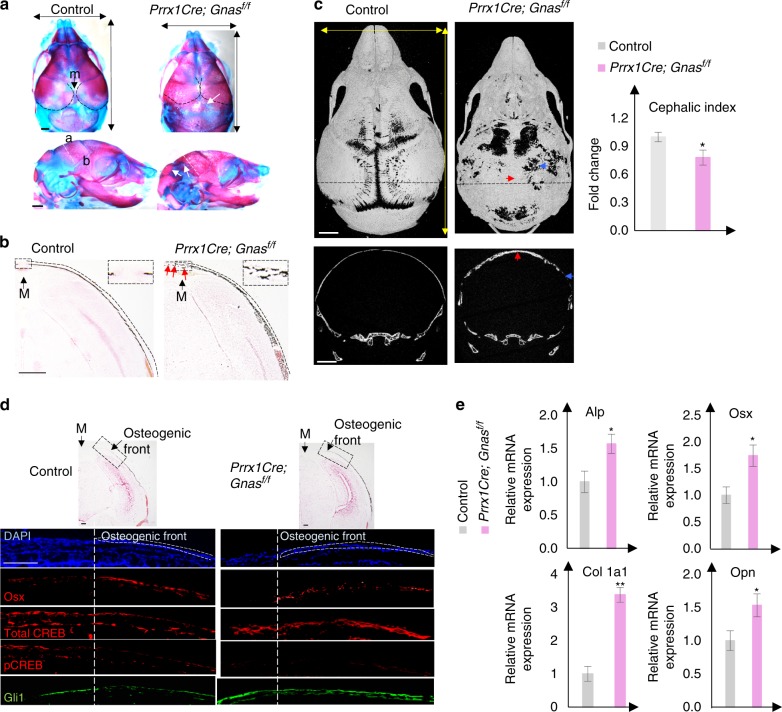
Loss of Gα_s_ accelerates intramembranous ossification. **a** Alizarin red and alcian blue staining of the mouse heads from E18.5 embryos. Accelerated ossification in the parietal bone (black dotted area), inter-parietal bone, and ectopic bone in between (white arrow) in the posterior fontanel are indicated. Upper: dorsal view. Lower: lateral view, position of coronal sections is indicated by a white dotted line. Maximum width and length of the skull (double arrows) are indicated. Scale bar: 0.5 mm. a apex, b base. **b** von Kossa and Safranin O staining of the parietal bone section (coronal section) at E18.5. M midline (arrow). Mineralized parietal region is indicated by a dotted line. Ectopic ossification in the suture is indicated (red arrows). Scale bar: 500 μm. **c** μCT image of the mouse heads from mice at P6. Upper panel: 3D reconstruction, dorsal view. Ectopic ossification (red arrow) and porous bone (blue arrow) are indicated. Lower panel: Coronal 2D view at the position indicated by the dotted line in 3D. Thickened bone (red arrow) and porous bone (blue arrow) are indicated. Scale bar: 1 mm. Quantitative analysis of cephalic index of the skull from P6 mice. Cephalic index = 100 × width/length. Results are shown as average measurements of three different mice±SD. *P < 0.05. **d** von Kossa and Safranin O staining of parietal bone from E15.5 embryos. The boxed osteogenic front regions were processed for immunofluorescent staining with the indicated antibodies. Images are shown in the lower panel. Osteogenic front is indicated (dotted line). Scale bar: 100 μm. **e** qRT-PCR analysis of osteoblast differentiation genes in the parietal bone tissues from P0 mice. Results are shown as average of three independent experiments±SD. *P < 0.05. **P < 0.01. The two-tailed Student’s *t* test was used in the statistic analysis

The change in cranial bone formation caused by Gα_s_ activation led us to test the hypothesis that *Gnas* removal in osteochondral progenitors may cause accelerated cranial bone formation in embryonic development and craniosynostosis after birth. We therefore removed *Gnas* in early osteochondral progenitors by generating the *Prrx1-Cre; Gnas*^*f/f*^ mice. Opposite to what we had found in the *Prrx1-Cre; Gnas*^*f(R201H)/+*^ mutants (Fig. 2), the *Prrx1-Cre; Gnas*^*f/f*^ mutants at E18.5 showed accelerated bone expansion from base to apex of the skull and increase in parietal and inter-parietal bone formation compared to the littermate control of *Prrx1-Cre; Gnas*^*f/+*^, *Gnas*^*f/f*^, or *Gnas*^*f/+*^ embryos or mice, which are phenotypically indistinguishable from the wild-type ones (Fig. 3a, b). Increased bone formation was also observed in the postnatal cranial bone leading to loss of sutures and therefore craniosynostosis (Fig. 3c). Premature suture closure may have led to reduced head width, though head length was also slightly reduced in the *Prrx1-Cre; Gnas*^*f/f*^ mutant mice compared to the controls (Fig. 3c). The CI was reduced compared to that of the control, suggesting that loss of Gα_s_ signaling may have caused a condition of dolichocephly, which can be a result of premature fusion of the sagittal suture. At the osteogenic front of the forming parietal bone at E15.5, Osx expression was accelerated and Gli1 expression was increased, but CREB phosphorylation was almost undetectable though total CREB levels were normal compared to the control (Fig. 3d, S6c). The increase in osteoblast differentiation was further confirmed by qRT-PCR. At P0, expression of *Alp*, *Osx*, *Col1a1*, and *Opn* were increased (Fig. 3e). Therefore, loss of Gα_s_ signaling by removing *Gnas* in early osteochondral progenitor cells activated Hh signaling and accelerated osteoblastic differentiation and ossification during cranial vault development. Importantly, although loss of Gα_s_ signaling accelerated osteoblast differentiation, the formed bone was of low quality with reduced mineral density and increased porosity (Fig. 3c, S3a, b). We found that this is likely due to increased osteoclast differentiation indicated by tartrate-resistant acid phosphatase staining and this defect could be caused by more pronounced reduction in the expression of *Osteoproteg*erin (*Opg*) compared to the *receptor activator of nuclear factor kappaB ligand* (*Rankl*) (Fig. S3c, d). While Rankl is required for osteoclast differentiation, its activity is physiologically counterbalanced by the decoy receptor Opg.^32–34^ Taken together, Gα_s_ signaling plays a key role in regulating osteoblast differentiation, maturation, and bone quality.

### Gα_s_ inhibits intramembranous ossification by inhibiting Hh signaling activity during cranial bone development

**Fig. 4:**
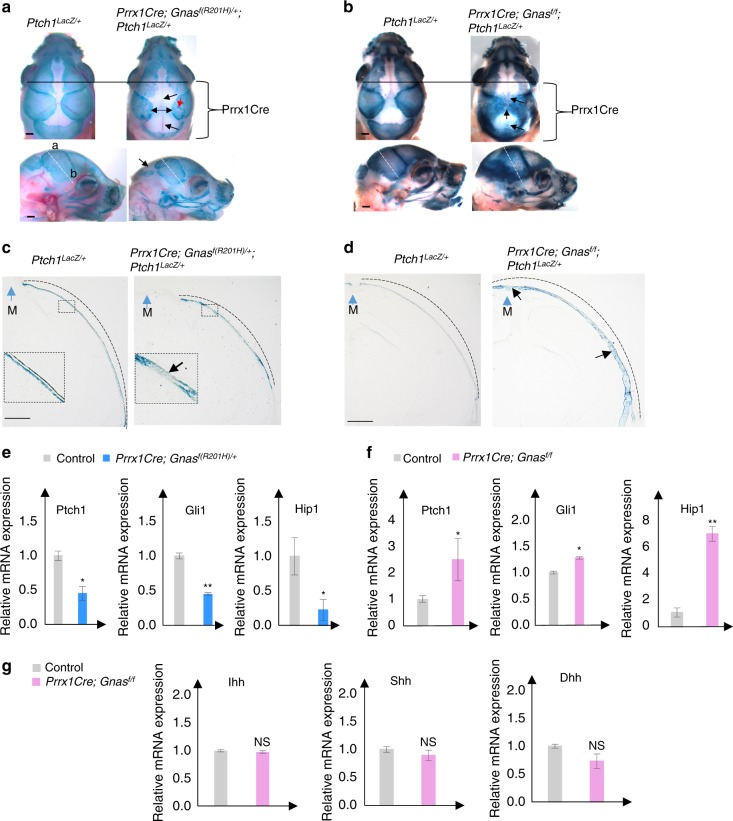
Gα_s_ regulates Hh signaling during cranial bone formation. **a**, **b** Whole-mount X-gal staining of the mouse heads from E18.5 embryos. *Prrx1Cre* expression regions are indicated. Upper: Dorsal view. Lower: Lateral view. Increased space devoid of X-gal staining or ectopic X-gal staining is shown (arrows). Islands of strong X-Gal staining are pointed by red arrows in **a**. Section position (white dotted line) is indicated. a apex, b base. Scale bar: 0.5 mm. **c**, **d** X-gal staining of cryostat sections from E18.5 embryos. M midline (arrow). The region with X-gal staining is shown by dotted line. Boxed regions in **c** are shown in higher magnification. Reduced (**c**) or ectopic and enhanced (**d**) Hh signaling is shown (black arrows). Scale bar: 0.5 mm. **e**–**g** qRT-PCR analysis of Hh signaling target gene (**e**, **f**) and Hh ligand gene (**g**) expression in the P0 parietal bone tissue. Results are shown as average of three independent experiments±SD. *P < 0.05, **P < 0.01. The two-tailed Student’s *t* test was used in the statistic analysis

Hh signaling is not only activated during cranial bone formation but is also functionally important for cranial bone development. Hh signaling activation caused by a *Ptch1* hypomorphic mutation led to craniofacial bone fusion,^35^ while reduced Hh signaling due to loss of *Gli2* function resulted in reduced intramembranous bone formation.^36^ We next asked whether Gα_s_ critically regulates intramembranous ossification during cranial vault bone formation by inhibiting Hh signaling. Upregulated Gli1 expression at the ossification front (Fig.1e) and its alteration by Gα_s_ signaling (Figs 2d and 3d) indicated that Hh signaling mediated Gα_s_ signaling activities in controlling intramembranous bone development. To further confirm this, we crossed the *Ptch1*^*LacZ/+*^ allele^19^ with the *Prrx1-Cre; Gnas*^*f(R201H)/+*^ or *Prrx1-Cre; Gnas*^*f/f*^ mutants and visualized Hh signaling in Gα_s_ gain- or loss-of-function mutant embryos, respectively, by X-Gal staining. In the nasal and anterior parts of frontal bones, where *Prrx1*Cre is not expressed, similar X-Gal staining pattern and intensities were found in the *Prrx1-Cre; Gnas*^*f(R201H)/+*^*;* *Ptch1*^*LacZ/+*^ mutants at E16.5 and E18.5 compared to the *Ptch1*^*LacZ/+*^ controls (Fig. 4a, S4a, c, e). However, in the posterior frontal bones, parietal, and inter-parietal bone areas where *Prrx1*Cre is expressed (Fig. S1a, b) as indicated by brackets in Fig. 4a, b, *Gnas*^*R201H*^ expression led to larger space devoid of X-Gal staining in the apex of the skull. In the forming parietal bone, X-Gal staining was also reduced in the mutant compared to the control though islands of strong X-Gal staining were found in the mutant at later stages (Fig. 4a, c). These island of cells might result from expansion of residual wild-type cells and/or wild-type reversal of mutant cells by epigenetic inactivation of the mutant *Gnas*^*R201H*^ allele, which can happen in the imprinted Gnas locus.^37,38^ These results indicate that Hh signaling is reduced by activated Gα_s_ signaling (Fig. 4a, c, Fig, S4a, c).

Conversely, in the *Prrx1-Cre;Gnas*^*f/f*^*;* *Ptch1*^*LacZ/+*^ mutant embryos at E16.5 and E18.5, X-gal staining of the parietal and inter-parietal bones was enhanced and ectopically detected in the sagittal suture and posterior fontanel (Fig. 4b, S4b). In addition, the forming parietal bone that was stained positive by X-Gal staining was expanded in size at E16.5 and E18.5 in the *Prrx1-Cre; Gnas*^*f/f*^*;* *Ptch1*^*LacZ/+*^ embryo compared to the *Ptch1*^*LacZ/+*^ control (Fig. 4d and S4d). These results indicate that Hh signaling is upregulated by loss of Gα_s_ signaling. Regulation of Hh signaling by Gα_s_ signaling in the cranial bone was further confirmed by qRT-PCR analysis of the parietal bone tissue from the P0 mouse pups as shown in Fig. S2a-c. While *Gnas*^*R201H*^ expression decreased the expression of Hh signaling target genes *Ptch1*, *Gli1*, and *Hip1*, loss of *Gnas* upregulated the expression of these Hh target genes (Fig. 4e, f). Importantly, expression of Hh ligand genes were not altered by loss of Gα_s_ signaling (Fig. 4g). Collectively, these data show that Gα_s_ signaling inhibits Hh signaling during cranial vault bone formation without altering Hh ligand expression.

**Fig. 5:**
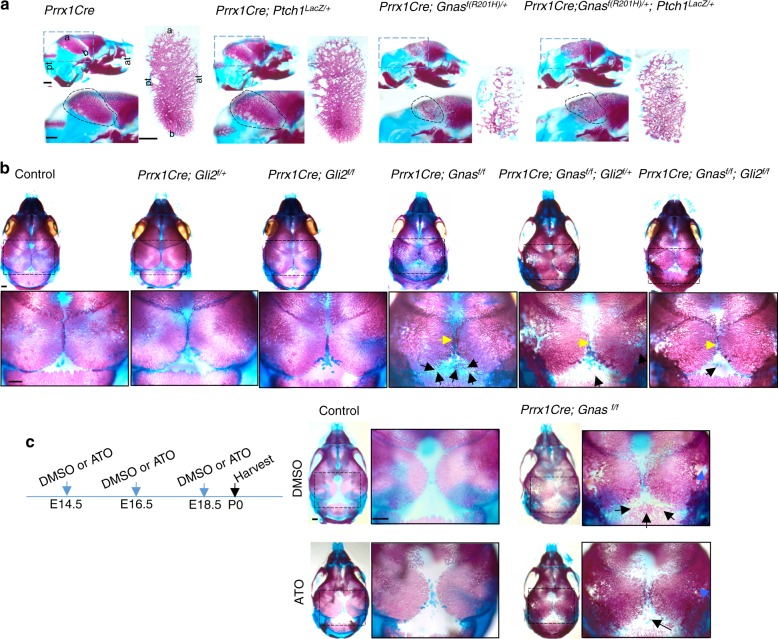
Increasing Hh signaling partially rescues the phenotypes caused by Gα_s_ signaling activation. **a** Alizarin red and alcian blue staining of the mouse heads from E16.5 embryos. Upper: lateral view. Lower: higher magnification view of the boxed area, parietal bone is circled. Right: The dissected parietal bone was flattened and shown in higher magnification. Orientation of skull (apex, base, anterior, posterior) is indicated in the first set of images. Scale bar; 0.5 mm. a apex, b base, pt posterior, at anterior. **b** Alizarin red and alcian blue staining of the mouse heads from P0 mice. Images were captured after removing mandibles and partial skull base. Upper: dorsal view. Lower: higher magnification view of the boxed region in the upper panel. Progressive rescue of accelerated ossification in the sagittal suture (yellow arrow) and posterior fontanel (black arrow) are shown. Scale bar; 0.5 mm. **c** The i.p. injection scheme of DMSO or ATO to pregnant mice is shown. P0 pups were harvested and analyzed by alizarin red and alcian blue staining. Black boxed regions are shown in higher magnification on the right. At posterior fontanels, ectopic ossification is shown by black arrows. Porous bone is shown by blue arrows. Scale bar: 0.5 mm

We next asked whether reduced Hh signaling mediated the effects of activated Gα_s_ signaling in intramembranous ossification during cranial vault bone formation. We reasoned that the inhibitory effects of Gα_s_ signaling in intramembranous ossification should be rescued by increasing Hh signaling if Hh signaling mediates the function of Gα_s_ signaling. We therefore generated the *Prrx1-Cre; Gnas*^*f(R201H)/+*^*; Ptch1*^*LacZ/+*^ mice as the *LacZ* insertion disrupts expression of *Ptch1*^19^ and Ptch1 is a negative regulatory component in the Hh signaling pathway.^39^ We found that, at E16.5, ossification in the developing parietal bone of the *Prrx1-Cre* and *Ptch1*^*LacZ/+*^ embryos was similar to the wild-type control. While ossification in the parietal bone of the *Prrx1-Cre; Gnas*^*f(R201H)/+*^ embryos was reduced compared to the *Prrx1-Cre* and *Ptch1*^*LacZ/+*^ controls, parietal bone formation was significantly rescued in the *Prrx1-Cre; Gnas*^*f(R201H)/+*^*; Ptch1*^*LacZ/+*^ embryos (Fig. 5a). Conversely, we genetically reduced Hh signaling in the *Prrx1-Cre; Gnas*^*f/f*^ mice by generating the *Prrx1-Cre; Gnas*^*f/f*^*;* *Gli2*^*f/+*^ and the *Prrx1-Cre; Gnas*^*f/f*^*;* *Gli2*^*f/f*^ mouse P0 pups (Fig. 5b). Gli2 is the major Gli transcription factor that activates downstream target gene expression in the Hh signaling pathway.^28^ Reduction of Gli2 led to mild reduction of cranial bone formation in the *Prrx1-Cre; Gli2*^*f/+*^ and *Prrx1-Cre; Gli2*^*f/f*^ mouse pups compared to the wild-type controls at P0 (Fig. 5b). Consistently, we found that increased ossification in the parietal and inter-parietal bones of the *Prrx1-Cre; Gnas*^*f/f*^ pups was progressively rescued in the sagittal suture and posterior fontanel regions of the *Prrx1-Cre; Gnas*^*f/f*^*;* *Gli2*^*f/+*^ and the *Prrx1-Cre; Gnas*^*f/f*^*;* *Gli2*^*f/f*^ pups at P0 (Fig. 5b). Furthermore, administration of a small molecule Gli inhibitor, arsenic trioxide (ATO),^40^ to pregnant female mice inhibited ectopic bone formation in the posterior fontanel region in the *Prrx1-Cre; Gnas*^*f/f*^ mouse pups at P0 (Fig. 5c). The rescuing effects are significant but not complete, which could be explained by at least two mechanisms. First, other Gli factors such as Gli3 also mediate transcription activities downstream of Hh signaling. Second, more than one signaling pathway is involved in *Gnas*-regulated cranial bone formation, for example, we also found that *Gnas* regulates Wnt/β-catenin in this manuscript and other studies.^2,5^ Taken together, our data indicate that Gα_s_ signaling inhibits cranial bone formation by inhibiting Hh signaling (Fig. 8d).

### Gα_s_ enhances Wnt/β-catenin signaling during intramembranous ossification of cranial bone development

**Fig. 6:**
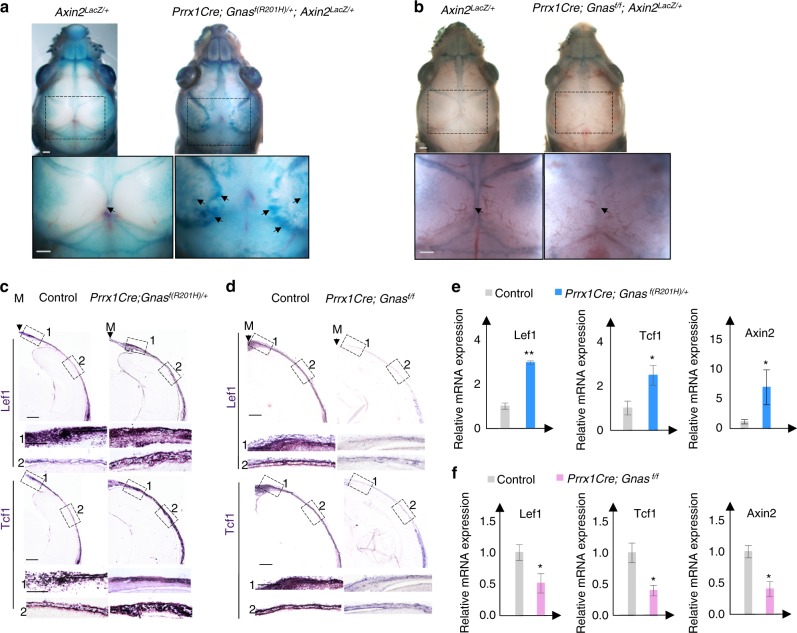
Gα_s_ regulates Wnt/β-catenin signaling during cranial vault formation. **a**, **b** Whole-mount X-gal staining of the mouse heads from P0 pups with the indicated genotypes. Boxed regions are shown in higher magnification in the lower panel. Enhanced (**a**) or reduced (**b**) X-gal staining at the osteogenic front and parietal bone is indicated (black arrow). Scale bar; 0.5 mm. **c**, **d** In situ hybridization using the probes of Wnt/β-catenin target genes Lef1 and Tcf1 on parietal bone sections from P0 pups. Osteogenic front (box 1) and matured bone (box 2) are shown in higher magnifications in the middle and bottom panels, respectively. Scale bar: 0.5 mm. **e**, **f** qRT-PCR analysis of Wnt/β-catenin signaling target gene expression in parietal bone tissues from P0 pups. Results are shown as average of three independent experiments±SD. *P < 0.05, **P < 0.01. The two-tailed Student’s *t* test was used in the statistic analysis

Gα_s_ signaling has been found to enhance Wnt/β-catenin signaling in the bone marrow stromal cells and during long bone development.^2,5,7^ We therefore tested whether such regulation also occurs during cranial bone formation. We examined Wnt/β-catenin signaling by X-gal staining using tissues from the *Axin2*^*LacZ/+*^ mice as *Axin2* is a direct transcription target of Wnt/β-catenin signaling^41–44^ (Fig. 6a). In the *Axin2*^*LacZ/+*^ control mice at P0, X-gal staining was detected in the parietal and inter-parietal bones and further upregulated in the forming sagittal suture and posterior fontanel (Fig. 6a, b). We generated the *Prrx1-Cre; Gnas*^*f(R201H)/+*^*; Axin2*^*LacZ/+*^ and *Prrx1-Cre; Gnas*^*f/f*^*;* *Axin2*^*L*acZ/+^ mutant mice and found that X-gal staining was enhanced in the developing parietal and inter-parietal bone, particularly in the osteogenic front by *Gnas*^*R201H*^ expression, but it was reduced by loss of *Gnas* compared to the controls (Fig. 6a, b). To further demonstrate that Wnt/β-catenin signaling is regulated by Gα_s_ signaling, we examined the expression of Wnt/β-catenin signaling target genes *Lef1* and *Tcf1* by performing in situ hybridization and qRT-PCR (Fig. 6c–e). Consistently, we found that Gα_s_ signaling activation by *Gnas*^*R201H*^ expression expanded expression domains of *Lef1* and*Tcf1* and enhanced their expression levels, while loss of Gα_s_ signaling reduced *Lef1* and*Tcf1* expression. These data indicate that Gα_s_ signaling also enhances Wnt/β-catenin signaling during cranial bone formation.

**Fig. 7:**
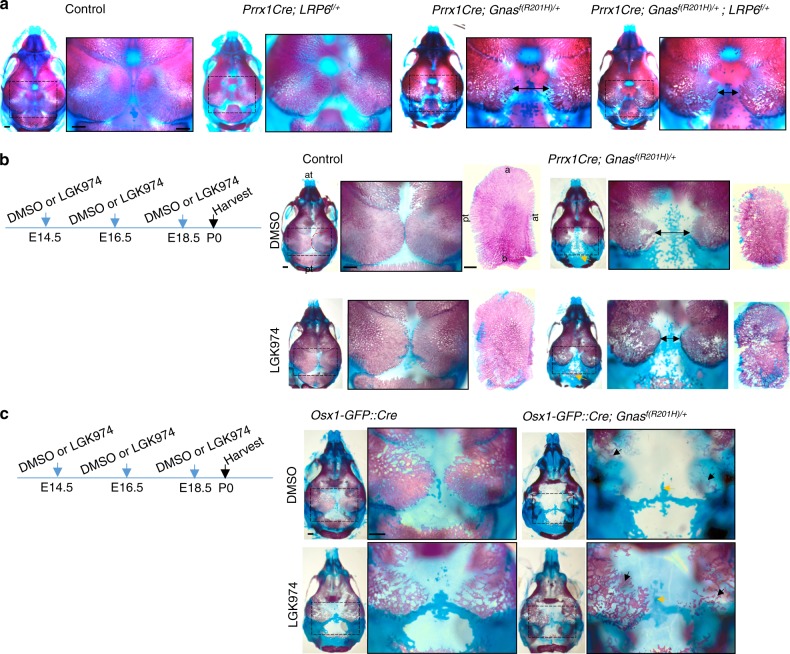
Decreased Wnt/β-catenin signaling partially rescues the phenotypes caused by Gα_s_ signaling activation. **a** Alizarin red and alcian blue staining of the mouse heads from P0 mouse pups with the indicated genotypes. Boxed areas are shown in higher magnifications on the right. Delayed ossification is indicated with wider suture in mutants and partial rescue is indicated by narrower suture (double arrows). Scale bar: 0.5 mm. **b**, **c** Alizarin red and alcian blue staining of the head from P0 pups with the indicated genotypes. Schemes of injection to the pregnant females (1 mg/kg LGK974 or DMSO) are shown. Boxed areas are shown in higher magnifications on the right. The parietal bone was dissected out and shown in higher magnification. Delayed ossification is indicated by larger suture space and higher bone porosity. Both were partially rescued by LGK974 treatment. Delayed cartilage dissolution is indicated (yellow arrow). Images were captured after removing mandibles and partial skull base. Scale bar: 0.5 mm

**Fig. 8:**
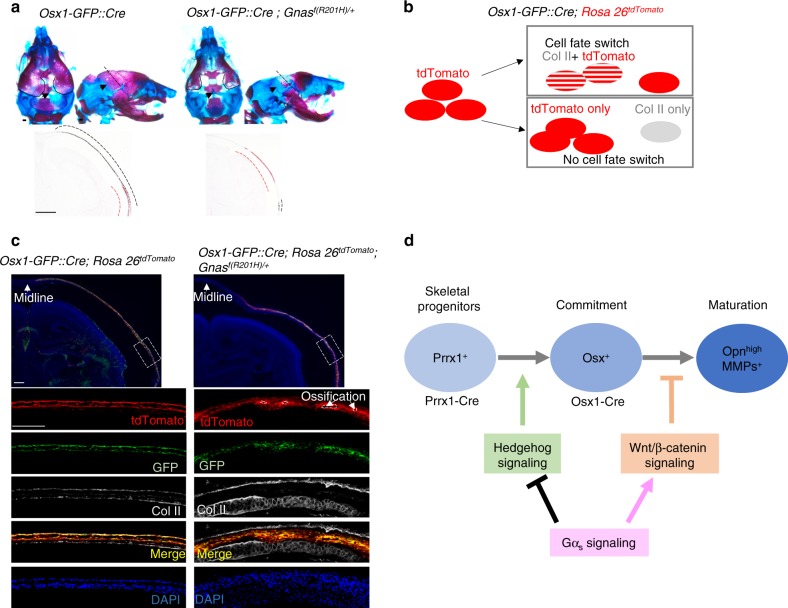
Activated Gα_s_ does not cause osteoblast to chondrocyte fate change. **a** Alizarin red and alcian blue staining of P0 mouse head with the indicated genotypes. Section position is indicated by dotted line on parietal bone. Coronal sections of the parietal bone showed increased cartilage (red dotted line) and decreased ossification (black dotted line) in the mutant after von Kossa and Safranin O staining (lower panel). Scale bar: 0.5 mm. **b** Schematics of lineage tracing of Osx-expressing cells and their descendants. **c** Lineage tracing in the Osx1-GFP::Cre; *Rosa 26*^*tdTomato*^ mice with immunofluorescence co-staining using the indicated antibodies on frozen sections from P0 pups with the indicated genotypes. Boxed regions are shown in higher magnification in the lower panel. Scale bar: 0.5 mm. **d** Schematics for the role of Gα_s_ signaling that controls skeletal progenitor cells commitment to osteoblast cells and osteoblast maturation during intramembranous ossification in cranial bone development. Gα_s_ inhibits Hedgehog signaling while promoting Wnt/β-catenin signaling. Hh signaling induces osteoblast commitment (Osx^+^) from progenitor cells (Prrx1^+^), whereas Wnt signaling inhibits osteoblast maturation (Opn^high^ and MMPs^+^)

Given the critical role of Wnt/β-catenin signaling in osteoblast differentiation, we then tested whether Wnt/β-catenin signaling also mediates the effects of Gα_s_ signaling in cranial bone formation. We hypothesized that reduction of Wnt/β-catenin signaling in the *Prrx1-Cre; Gnas*^*f(R201H)/+*^ mice should ameliorate the defects of cranial bone formation. We therefore reduced Wnt/β-catenin signaling activity in mesenchymal progenitor cells by generating the *Prrx1Cre; Gnas*^*f(R201H)/+*^*; Lrp6*^*f*/+^ mice^45^ (Fig. 7a). Lrp6 is a Wnt co-receptor dedicated to the Wnt/β-catenin signaling pathway.^46,47^ Interestingly, although Gα_s_ activation and reduction of *Lrp6* in *Prrx1Cre*-expressing cells both lead to reduced cranial bone formation compared to the control (Fig. 7a), cranial bone formation was increased in the *Prrx1Cre; Gnas*^*f(R201H)/+*^*; Lrp6*^f/+^ mice at P0. These results are consistent with our observation in the long bones.^5^ Furthermore, we found that administering LGK974, an inhibitor of Wnt secretion,^48^ delayed ossification during cranial bone formation in the wild-type controls (Fig. 7a, b). However, although cranial bone formation was delayed in both *Prrx1-Cre; Gnas*^*f(R201H)/+*^ and *Osx1-GFP::Cre; Gnas*^*f(R201H)/+*^ mutants (Fig. 7b, c), LGK974 administration to pregnant females rescued cranial bone formation in both *Prrx1-Cre; Gnas*^*f(R201H)/+*^ and *Osx1-GFP::Cre; Gnas*^*f(R201H)/+*^ mutants (Fig. 7b, c). Taken together, we found that Gα_s_ signaling activation in the mesenchymal progenitor cells or early osteoblast cells both led to activated Wnt/β-catenin signaling, which delayed ossification (Fig. 8d).

### Gα_s_ signaling activation in osteoblast lineage increases cartilage formation without changing cell fate specification

In both *Prrx1-Cre; Gnas*^*f(R201H)/+*^ and *Osx1-GFP::Cre; Gnas*^*f(R201H)/+*^ mice, we found that reduced ossification in the cranial bone was accompanied by increased cartilage formation as evidence by increased Safranin O staining compared to the controls (Fig. 8a, S5a). It has been shown that osteoblast cells and chondrocytes are specified from their common osteochondral progenitors and loss of *Osx* or Wnt/β-catenin signaling leads to chondrocyte instead of osteoblast differentiation.^27,49^ We therefore asked whether increased cartilage formation was due to transdifferentiation of Osx^+^ cells to chondrocytes when Gα_s_ signaling was activated in the *Osx1-GFP::Cre; Gnas*^*f(R201H)/+*^ mice. The Osx^+^ cells and their descendants were traced by TdTomato expression in the *Osx1-GFP::Cre; Rosa 26*^*tdTomato*^ mice.^50^ As the *Osx1-GFP::Cre* line expresses a Cre::GFP fusion protein,^18^ osteoblasts are marked by green fluorescent protein (GFP) expression. Chondrocytes were detected by immunofluorescence staining with Collagen type II (ColII) antibodies. We reasoned that, if there is a cell fate switch from osteoblasts to chondrocytes, some of the tdTomato^+^ cells should also express ColII, otherwise there should be no overlap between tdTomato^+^ and ColII^+^ cells (Fig. 8b). In the *Osx1-GFP::Cre; Gnas*^*f(R201H)/+*^*; Rosa 26*^*tdTomato*^ mice, while there was large overlap between tdTomato and GFP expression, a few tdTomato^+^ cells lost GFP expression. However, none of these cells were ColII^+^, indicating that the expansion of the ColII^+^ cell population is not a result of transdifferentiation of Osx^+^ cells (Fig. 8c, S6d). Interestingly, just as loss of *Osx* led to osteoblast conversion to chondrocytes, loss of Smo or β-catenin in early osteochondral progenitors during endochondral bone formation also led to similar osteoblasts to chondrocytes' cell fate changes.^21,49,51,52^ However, although Gα_s_ signaling activation in Osx^+^ cells during intramembranous ossification resulted in expanded cartilage formation and reduced Hh signaling (Fig. 4a, c, e), we found that expanded cartilage is not derived from Osx^+^ cells in the *Osx1-GFP::Cre; Gnas*^*f(R201H)/+*^*; Rosa 26*^*tdTomato*^ mice at P0(Fig. 8c). The cartilage found in the *Osx1-GFP::Cre; Gnas*^*f(R201H)/+*^ mutant skull looked similar to the one reported in the *Membrane-type 1 matrix metalloproteinase* (*Mt1-mmp* or *Mmp14*) mutant mice,^53^ suggesting that the defect is in the cartilage remodeling rather than cell fate determination. Consistent with this notion, we found that, associated with altered bone formation in the *Prrx1Cre; Gnas*^*f(R201H)/+*^ and *Prrx1Cre; Gnas*^*f/f*^ mice, cartilage remodeling in the skull was also changed (Fig. S5a, b). To further confirm this, we examined the expression of *Mt1-mmp* and *Mmp13* and found both were reduced in the *Prrx1Cre; Gnas*^*f(R201H)/+*^ mutant parietal bone but increased in the *Prrx1Cre; Gnas*^*f/f*^ mice compared to the control (Fig. S5c, d).

Taken together, our data show that proper control of Gα_s_ signaling is required to ensure normal cranial bone formation by regulating both Hh and Wnt signaling and reduction of Gα_s_ signaling contributes to Hh signaling activation during normal intramembranous ossification, but not endochondral ossification. These results provide further insights in strategic development to treat FD, POH, and other related craniofacial bone malformation such as craniosynostosis.

## Discussion

In this study, we have identified critical roles of Gα_s_ signaling in regulating normal intramembranous ossification during cranial bone development. Both gain- or loss-of-function mutations in *GNAS* have been found to cause severe cranial bone defects in MAS or POH human patients, respectively.^8,9^ Our findings in this study support a model in which normal intramembranous bone development shares common underlying cellular and molecular mechanisms with ectopic intramembranous bone formation such as the one in POH. This model provides a new conceptual framework to further identify basic regulatory mechanisms of normal bone formation in the cranium and test the contribution of bone development factors in ectopic bone formation and expansion under pathological conditions. The knowledge gained in these studies will facilitate development of therapeutic approaches for craniosynostosis, cleidocranial dysplasia, POH, and acquired HO.

There are major gaps in understanding the molecular mechanisms that distinguish intramembranous ossification versus endochondral ossification. We found here that regulation of Hh signaling exhibits major difference between long bone development in the limb and flat bone formation in the skull. We have shown previously that Hh signaling is required before Wnt/β-catenin signaling during osteoblast differentiation.^19^ It has been well established that ligand-dependent Hh signaling is absolutely required for endochondral bone formation. In the absence of Ihh or Hh receptor Smo, osteoblast cells cannot form during endochondral ossification.^20,21^ Therefore, one critical function of preformed cartilage during endochondral bone formation is to provide Ihh required for osteoblast differentiation that first occurs in perichondrium adjacent to the pre-hypertrophic chondrocyte region. While Hh signaling is also activated during intramembranous ossification during cranial bone formation, surprisingly, Ihh and even Smo that mediates all ligand-dependent Hh signaling are not required for osteoblast differentiation. Consistent with previous findings in ectopic bone formation under pathological conditions that Gα_s_ signaling inhibits Hh signaling downstream of Smo,^1^ we found in this study that, during normal cranial bone formation, Gα_s_ signaling plays a prominent role contributing to Hh signaling regulation in a ligand-independent manner during normal intramembranous bone formation. The parathyroid hormone-related peptide (PTHrP)/PTH receptor PTH1R is predominantly coupled to Gα_s_^54,55^ and plays important roles regulating osteoblast differentiation. It has been shown that, at P0, the *Prrx1Cre; PTH1R*^*fl/fl*^ mice exhibited greatly enhanced mineralization in limbs and calvaria,^56^ thus PTH1R may be one of the GPCRs that regulate Gα_s_ signaling during cranial bone formation. PTH and locally produced PTHrP have pro-differentiating and pro-survival effects during bone formation. However, as continuous exposure to PTH reduces osteogenic differentiation,^57–59^ sustained PTH1R/Gα_s_ signaling activation inhibits osteoblast maturation in the long bone.^5,6^ Importantly, the function of PTH1R/Gα_s_ signaling in endochondral bone formation is also mediated by its role in inhibiting chondrocyte hypertrophy of the growth plates,^5,60–64^ where *Ihh* is expressed in pre-hypertrophic chondrocytes. Therefore, an important difference between endochondral versus intramembranous bone formation is that PTH1R/Gα_s_ signaling also indirectly inhibits Hh signaling by delaying *Ihh* expression in chondrocytes during endochondral bone formation. The dual roles of *Ihh* in controlling chondrocyte hypertrophy and osteoblast differentiation indicate that *Ihh* expression couples chondrocyte proliferation and hypertrophy with osteoblast differentiation from mesenchymal progenitors in the perichondrium during endochondral ossification. During cranial bone formation, however, the role of *Ihh* expression at the osteogenic front in osteoblastic induction is limited, and other signaling pathways such as Gα_s_ signaling also control intramembranous ossification by regulating Hh signaling activities downstream of Smo. Therefore, the findings in this study provide a new conceptual framework to further understand the regulatory circuitry of craniofacial bone formation.

Hh signaling can be regulated by Hh ligand and other signaling component like Gα_s_. Bone can normally form when Hh and Wnt/β-catenin signaling levels are maintained in a certain range. Loss of *Smo* in the Prrx1^+^ mesenchymal cells blocked ligand-dependent Hh signaling, while the Gα_s_-regulated Hh or Wnt/β-catenin signaling is still intact. The mild bone phenotypes in the *Prrx1Cre; Smo*^*f/f*^ embryo allowed us to conclude that ligand-independent Hh signaling such as the one regulated by Gα_s_ played significant roles in bone formation in the *Prrx1*-targeted skull tissues. Activated Gα_s_ signaling in the *Prrx1Cre;Gnas*^*f(201H)/+*^ mice not only reduced Hh signaling but also activated Wnt/β-catenin signaling in progenitor cells, and both led to reduced bone formation. It is foreseeable that abnormally activated Wnt/β-catenin signaling sensitized defects caused by Hh signaling reduction. Because of this, increasing Hh signaling by reducing Ptch1 will bring Hh signaling to a level closer to the normal one, thus reducing the phenotypic severity. Furthermore, Wnt/β-catenin signaling levels higher or lower than the normal range reduce bone formation. *Gnas* gain-of-function mutation causes reduced bone formation partially by enhancing Wnt/β-catenin signaling to levels above the normal range in the wild-type embryo. Therefore, reduction of Wnt/β-catenin signaling by deleting *Lrp6* or administrating LGK974, which reduce bone formation in a wild-type background, brought down the Wnt/β-catenin signaling levels in the *Prrx1Cre;Gnas*^*f(201H)/+*^ mutant closer to the normal range and thus the mutant phenotypes were rescued.

Gα_s_ signaling regulates osteoblast differentiation by regulating both Wnt/β-catenin and Hh signaling. As we have shown in our previous studies,^1,2,5^ the effect of gain or loss of Gα_s_ signaling in osteoblast differentiation was mediated predominantly by Wnt/β-catenin or Hh signaling, respectively. While Wnt/β-catenin signaling shown by the Axin2-LacZ expression was reduced in the loss of Gα_s_ signaling mutant (Fig. 6b), the bone phenotypes were mainly driven by elevated Hh signaling caused by Gα_s_ loss (Fig. 4b) even if Wnt/β-catenin signaling is reduced at the same time. However, the functional impacts of Gα_s_ regulation of Wnt/β-catenin signaling is also different in intramembranous versus endochondral ossification. It appears that Gα_s_ regulates endochondral ossification mainly through Wnt/β-catenin signaling while its role in osteoblast induction during intramembranous ossification is mainly determined by its regulation of Hh signaling. This explains the phenotypic discrepancy of bone formation caused by Gα_s_ signaling activation in Osx^+^ cells, which showed increased endochondral bone formation^5^ while intramembranous ossification is severely inhibited with reduced cartilage dissolution (Fig. 8a).

The less severe phenotypes of *Prrx1Cre* mutant than the *OsxCre*-mediated *Gnas* mutants (Fig.7b, c) are likely due to the different spatial and temporal onset of the *Cre* expression. *Prrx1Cre* targets earlier mesenchymal progenitor cells before Osx expression but mostly from the mesodermal origin, while *OsxCre* targets all osteoblast lineage cells regardless of their mesodermal or NC origins. As the gain-of-function *Gnas* promotes Wnt/β-catenin signaling, which is known to stimulate progenitor cell proliferation and reduce osteoblast maturation, *Prrx1Cre*-targeted mutation may lead to expansion of progenitor cells but reduced osteoblast maturation as has been shown in our studies of the *Sox9CreER*-mediated *Gnas* mutant.^5^ In the *OsxCre; Gnas*^*f(R201H)/+*^ mutant, there should be less progenitor cell proliferation due to the later onset of the *Cre* but more broadly affected skull bone tissues, leading to more severe phenotypes of reduced bone ossification.

The mouse models we have established allowed us to test small molecules that target Hh or Wnt signaling to ameliorate bone phenotypes caused by disrupted Gα_s_ signaling. Craniofacial hyperostosis/FD in MAS or craniosynostosis/POH are still unmet medical challenges. The Hh and Wnt signaling pathways act downstream of Gα_s_ signaling and both are important drug targets in many other diseases, including cancer. Therefore, some of the drugs already developed targeting Hh or Wnt signaling can be repurposed to treat bone defects including those in the craniofacial regions of FD or POH patients. Furthermore, our studies highlight the unparalleled opportunities offered by human genetic diseases in revealing fundamentally important genes and pathways in both human biology and pathology. Therefore, not only understanding the pathological mechanisms of genetic disease like POH and FD have allowed us to uncover a fundamental mechanism underlying a physiological bone formation process, the precise connection of pathological bone formation with normal bone development will provide invaluable insights in refining disease diagnosis and development of treatment strategies. The mechanistic investigation of cranial bone development in FD and POH models has significantly expanded previous knowledge of Wnt and Hh signal transduction in osteoblast differentiation and may provide new insights in other craniofacial bone diseases with similar defects, such as craniometaphyseal dysplasia and craniodiaphyseal dysplasia.

## Materials and methods

### Mouse lines

All animal experiments were carried out according to protocols approved by the Harvard Medical School Institutional Animal Care and Use Committee. All mice have been described in the literature: *Gnas*^*f/f*^,^29^ *Prrx1Cre*,^24^ *Gnas*^*f(R201H)/+*^,^5^ *Osx1-GFP::Cre(Osx Cre)*,^18^ *Lrp6*^*f*/+^,^45^ *Ptch1*^*LacZ/+*^,^19^ *Smo*^*f/f*^,^65^ and *Gli2*^*f/f*^.^66^ The *Axin2*^*LacZ/+*^ mice^42^ were purchased from the Jackson Laboratories. Both male and female embryos were used in the analyses as sex could not be clearly identified in embryos or neonatal mice. Three or more littermate groups were examined and representative images are shown in figures.

### Skeletal preparation

Alcian blue staining for cartilage and Alizarin red staining for mineralized tissues were performed as described.^67^ Briefly, embryos and early-postnatal mouse pups were collected and skinned carefully. Internal organs were then eviscerated and the embryos were placed in 100% ethanol overnight. Staining was performed according to a previously described protocol.^18^ Some dorsal views of the skull were captured after removing mandibles and partial skull base (Fig S2d-e).

### von Kossa staining

Cryostat sections were stained with 1% silver nitrate solution under a 60 W lamp for 1 h. Slides were rinsed three times in distilled water. Sodium thiosulfate 5% was added to the slides for 5 min. Slides were rinsed three times in distilled water and counterstained with 1% Safranin O or fast red for 5 min. Slides were rinsed three times in 1% acetic acid or distilled water before mounted in mounting medium.

### Histological and immunohistochemistry staining

Embryos and early-postnatal specimens were fixed in 4% (wt/vol) paraformaldehyde in phosphate-buffered saline (PBS). For immunohistochemistry, cryostat sections were blocked in 5% donkey serum and 0.3% Triton X-100 in PBS and performed with the following primary antibodies: rabbit anti-Osx (#sc-22536, Santa Cruz Biotechnology) 1:500, rabbit anti-CREB1 (#sc-25785, Santa Cruz Biotechnology) 1:100, rabbit anti-pCREB (#06-519, Millipore) 1:100, goat anti-Gli1 (#sc-6153, Santa Cruz Biotechnology) 1:50, and goat anti-ColII (#sc-7764, Santa Cruz Biotechnology) 1:100. Secondary antibodies are: Alexa Fluor® 488 donkey anti-goat (#A11055, Life Technologies), Alexa Fluor® 568 donkey anti-rabbit (#A10042, Life Technologies), and Alexa Fluor® 647 donkey anti-goat (#A21447, Life Technologies). Signals of 647 were read by Cy5 filter and the signal is artificially shown in white color. Cryostat sections were mounted in mounting medium with 4,6-diamidino-2-phenylindole from Vector laboratories (H-1200).

### RNA in situ hybridization

RNA in situ hybridization was performed using digoxygenin-labeled anti-sense RNA probes as described before.^68^

### X-gal staining

Embryos were fixed in 0.5% formaldehyde and 0.1% glutaraldehyde and X-gal staining was performed according to previously described procedures.^69^

### Quantitative real-time PCR

Total RNA from mouse calvarial bone tissue (Fig. S2a-c) was prepared using the TRIZOL reagent (Life Technologies) or RNeasy Mini Kit (Qiagen) according to the manufacturer’s protocols. cDNA was synthesized from total RNA (1–3 μg) using SuperScript II Reverse Transcriptase with random primer (Life Technologies). qRT-PCR were performed using SYBR Select Master Mix on StepOnePlus thermal cycler from Applied Biosystems. Expression levels were always given relative to glyceraldehyde 3-phosphate dehydrogenase (GAPDH).

GAPDH: Forward 5′-AGGTCGGTGTGAACGGATTTG-3′, Reverse 5′-TGTAGACCATGTAGTTGAGGTCA-3′;

Alk Phos: Forward 5′-CTTGACTGTGGTTACTGCTGAT-3′, Reverse 5′-GGAATGTAGTTCTGCTCATGGA-3′;

Osx: Forward 5′-CCCACTGGCTCCTCGGTTCTCTCC-3′, Reverse 5′-GCTBGAAAGGTCAGCGTATGGCTTC-3′;

Col1a1: Forward 5′-CACCCTCAAGAGCCTGAGTC-3′, Reverse 5′-GTTCGGGCTGATGTACCAGT-3′;

Opn: Forward 5′-GGCATTGCCTCCTCCCTC-3′, Reverse 5′-GCAGGCTGTAAAGCTTCTCC-3′;

Lef1: Forward 5′-CTTCGCCGAGATCAGTCATCC -3′, Reverse 5′-ACGGGTCGCTGTTCATATTGG-3′;

Tcf1: Forward 5′-TCGAGAAGAGCAGGCCAAGT-3′, Reverse 5′-AGAGCACTGTCATCGGAAGGAA-3′;

Axin2: Forward 5′-CCATTGGAGTCTGCCTGTG-3′, Reverse 5′-GGACACTTGCCAGTTTCTTTG-3′;

Ptch1: Forward 5′-ACCCGTCAGAAGATAGGAGAAG-3′, Reverse 5′-TTCCCAGAAGCAGTCCAAAGG-3′;

Gli1: Forward 5′-AATCCAATGACTCCACCACAAG -3′, Reverse 5′-GTCGAGGCTGGCATCAGAA-3′;

Hhip: Forward 5′-GGGAAAAACAGGTCATCAGC-3′, Reverse 5′-ATCCACCAACCAAAGGGC-3′;

Ihh: Forward 5′-ACGTGCATTGCTCTGTCAAGT-3′, Reverse 5′-CTGGAAAGCTCTCAGCCGGTT-3′;

Shh: Forward 5′-GATGACTCAGAGGTGCAAAGACAA-3′, Reverse 5′-TGGTTCATCACAGAGATGGCC-3′;

Dhh: Forward 5′-CCCGACATAATCTTCAAGGATGA-3′, Reverse 5′-GCGATGGCTAGAGCGTTCAC-3′;

Mmp13: Forward 5′-TTCTGGTCTTCTGGCACACGCTTT-3′, Reverse 5′-CCAAGCTCATGGGCAGCAACAATA-3′;

Mt1-mmp: Forward 5′-CTGCCTACGAGAGGAAGGATGG-3′, Reverse 5′-ATTCAGGGCAGTGGACAGCG-3′.

### Small molecule treatment

ATO 5 mg (Sigma Aldrich 202673-5 G) was dissolved in 15 mL conical tube with 1 mL of 1 N NaOH. Autoclaved PBS 7 mL was then added to the tube. HCl 1.2 N was used to adjust the pH to 7.2, and PBS was added to make up the volume to 10 mL. LGK974 (Toronto Research Chemicals L397640) was dissolved in DMSO and then diluted with corn oil before injection. Pregnant females were injected with ATO or LGK974 by intraperitoneal injection at a concentration of 5 mgkg^−1^ or 1 mgkg^−1^, respectively, every other day. Equivalent volumes of vehicle were injected as control. Mice were injected with care with ATO during their pregnancy starting at E13.5 or LGK974 at E14.5.

### Statistical analysis

Statistical significance was assessed using two-tailed Student’s *t* test for comparisons between two groups. *P* values <0.05 were considered significant. Data are presented as mean±standard deviations (SD) unless otherwise indicated.

## Electronic supplementary material

Lineage tracing the Prrx1-Cre targeted cells in the developing skull

Schematics of skull tissue preparations

Loss of Gαs in the cranial bone caused bone loss with increased osteoclast numbers

Gαs regulates Hh signaling during cranial bone formation

Gαs signaling regulates cartilage dissolution during skull development

Quantification of immunostaining at osteogenic front at E15.5 and P0
