# Supplementary figures and images for: Gαs signaling controls intramembranous ossification during cranial bone development by regulating both Hedgehog and Wnt/β-catenin signaling
Source: Bone Res. 2018 Nov 20;6:33. doi: 10.1038/s41413-018-0034-7 (PMC6242855; doi:10.1038/s41413-018-0034-7)

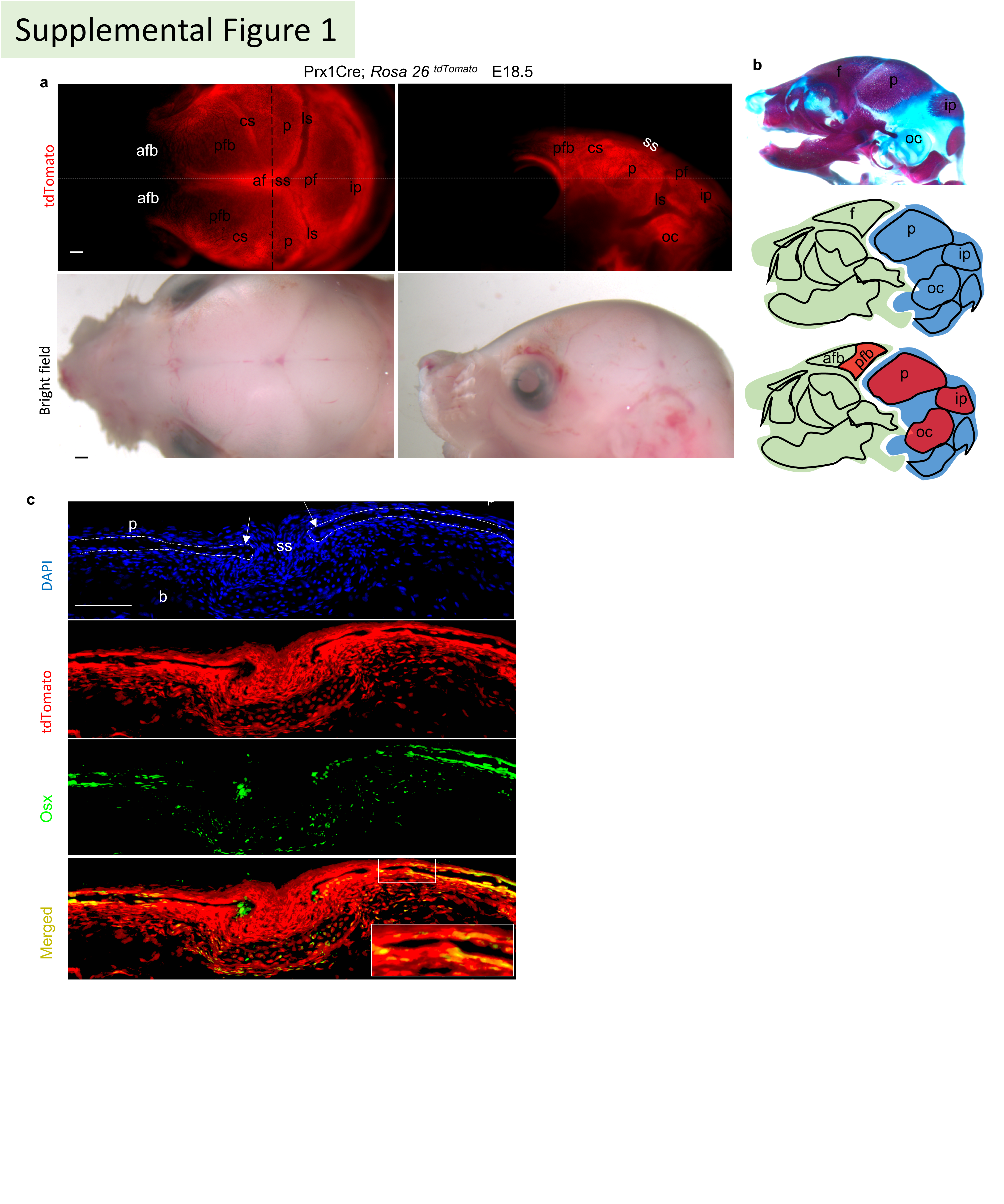

Supplement: Supplementary file 1 — Lineage tracing the Prrx1-Cre targeted cells in the developing skull [file 41413_2018_34_MOESM1_ESM.tif]

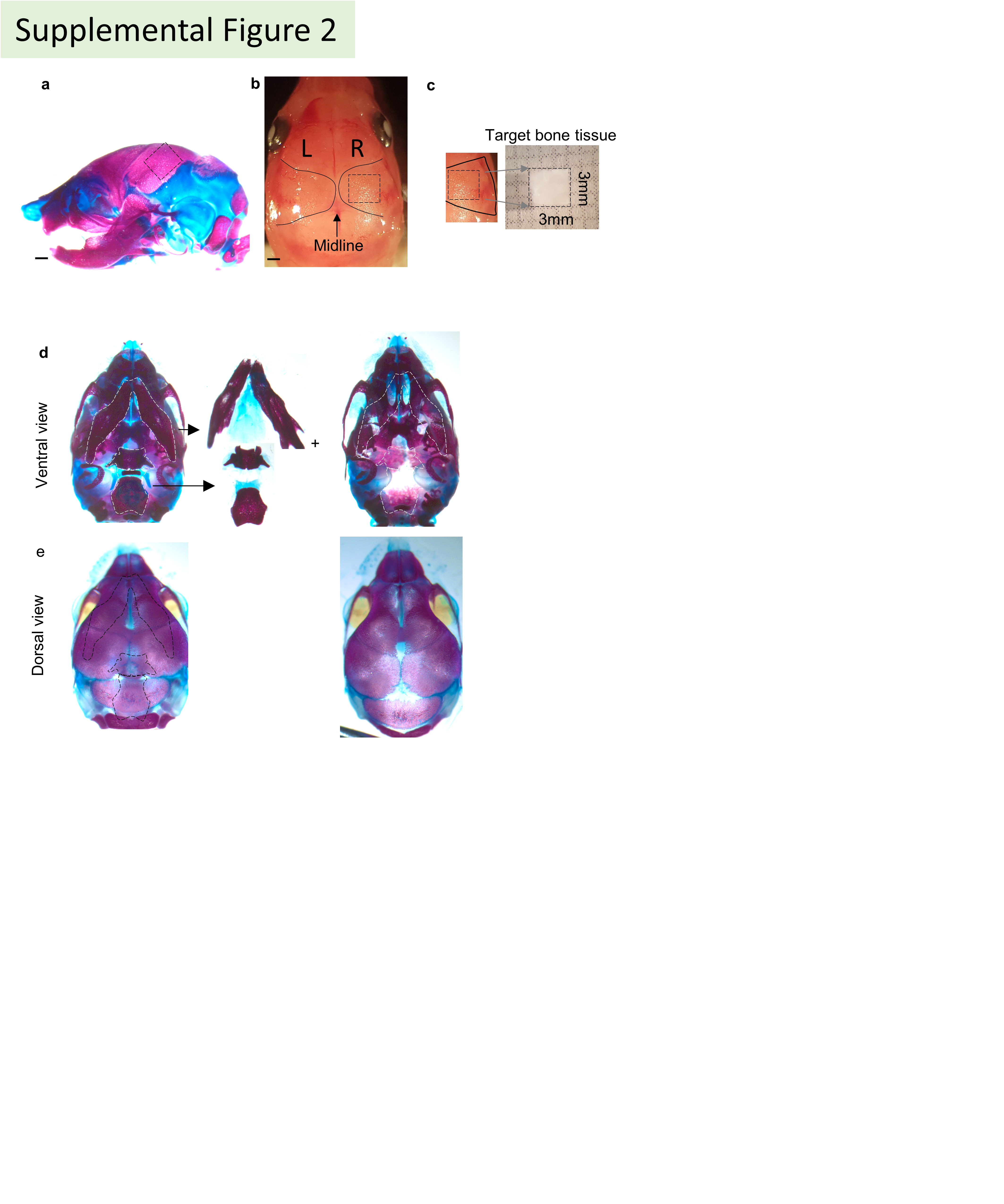

Supplement: Supplementary file 2 — Schematics of skull tissue preparations [file 41413_2018_34_MOESM2_ESM.tif]

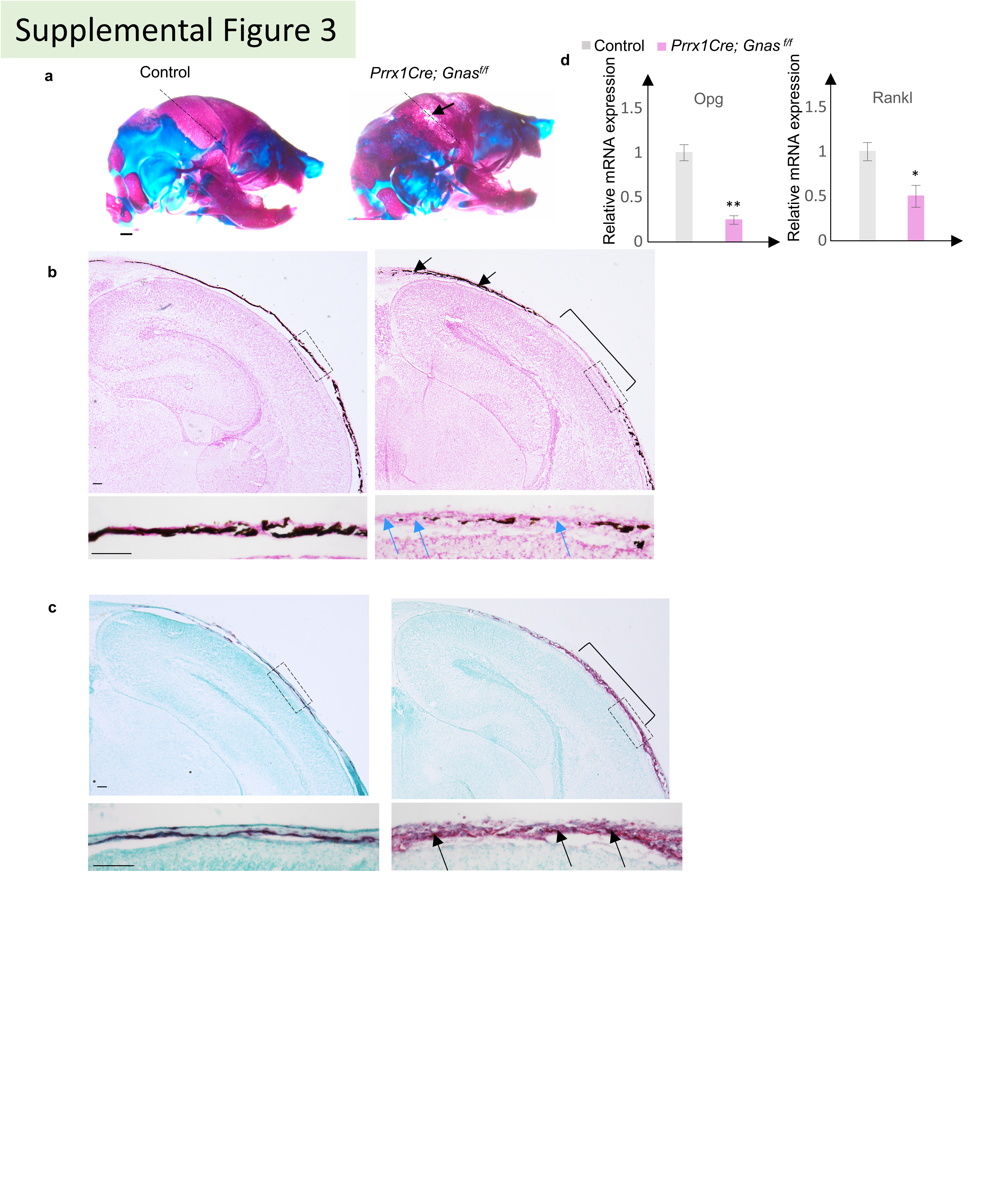

Supplement: Supplementary file 3 — Loss of Gαs in the cranial bone caused bone loss with increased osteoclast numbers [file 41413_2018_34_MOESM3_ESM.tif]

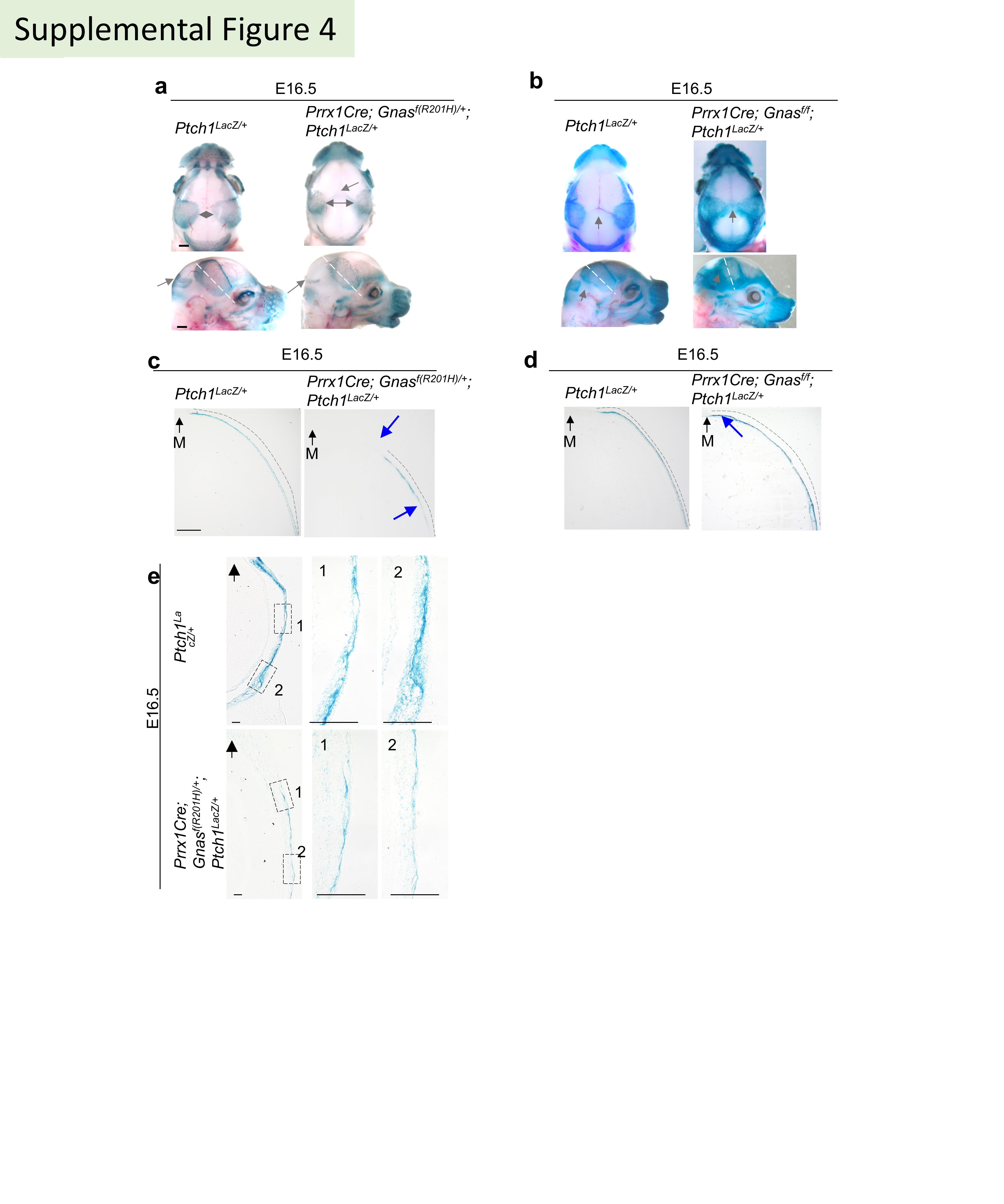

Supplement: Supplementary file 4 — Gαs regulates Hh signaling during cranial bone formation [file 41413_2018_34_MOESM4_ESM.tif]

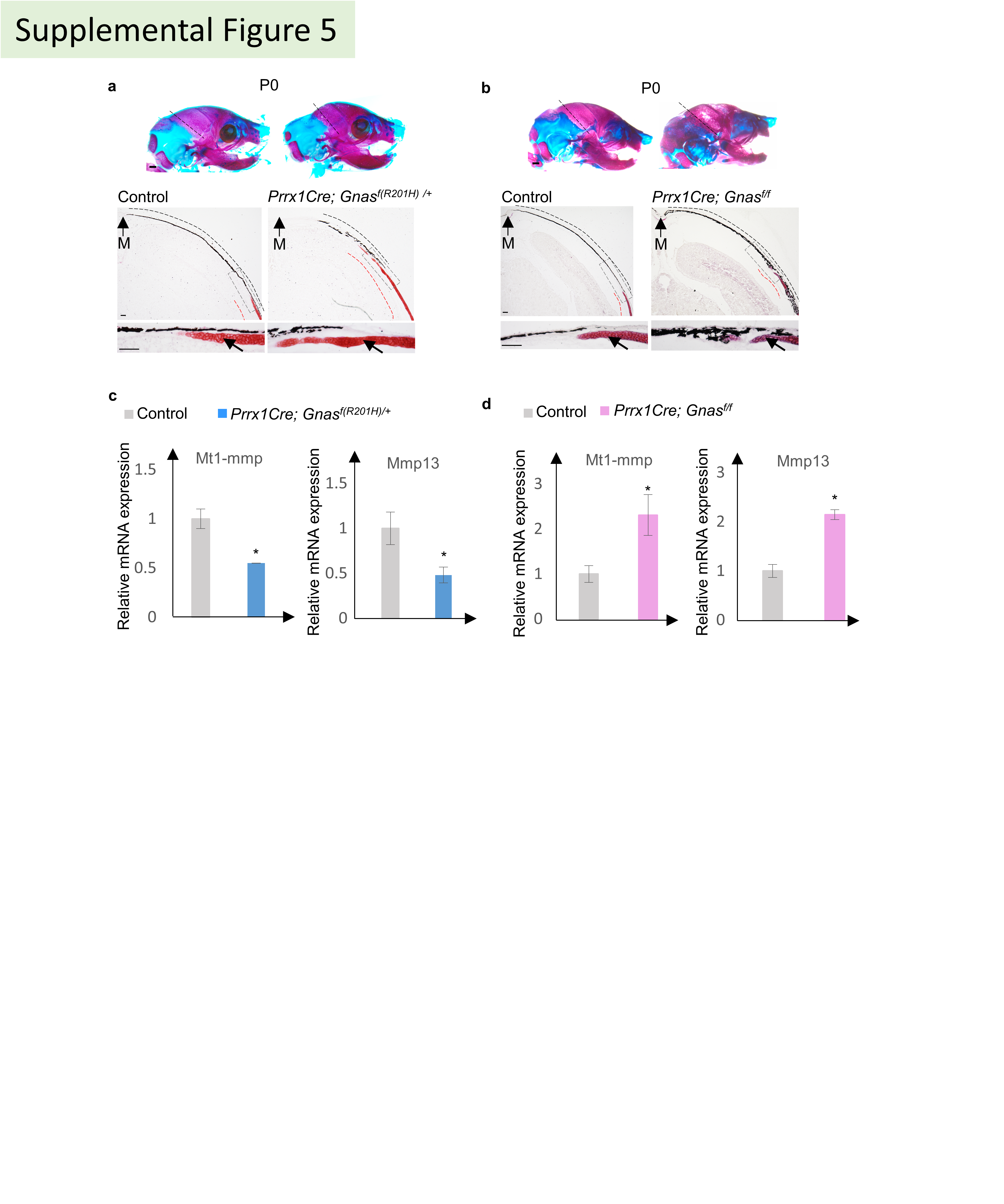

Supplement: Supplementary file 5 — Gαs signaling regulates cartilage dissolution during skull development [file 41413_2018_34_MOESM5_ESM.tif]

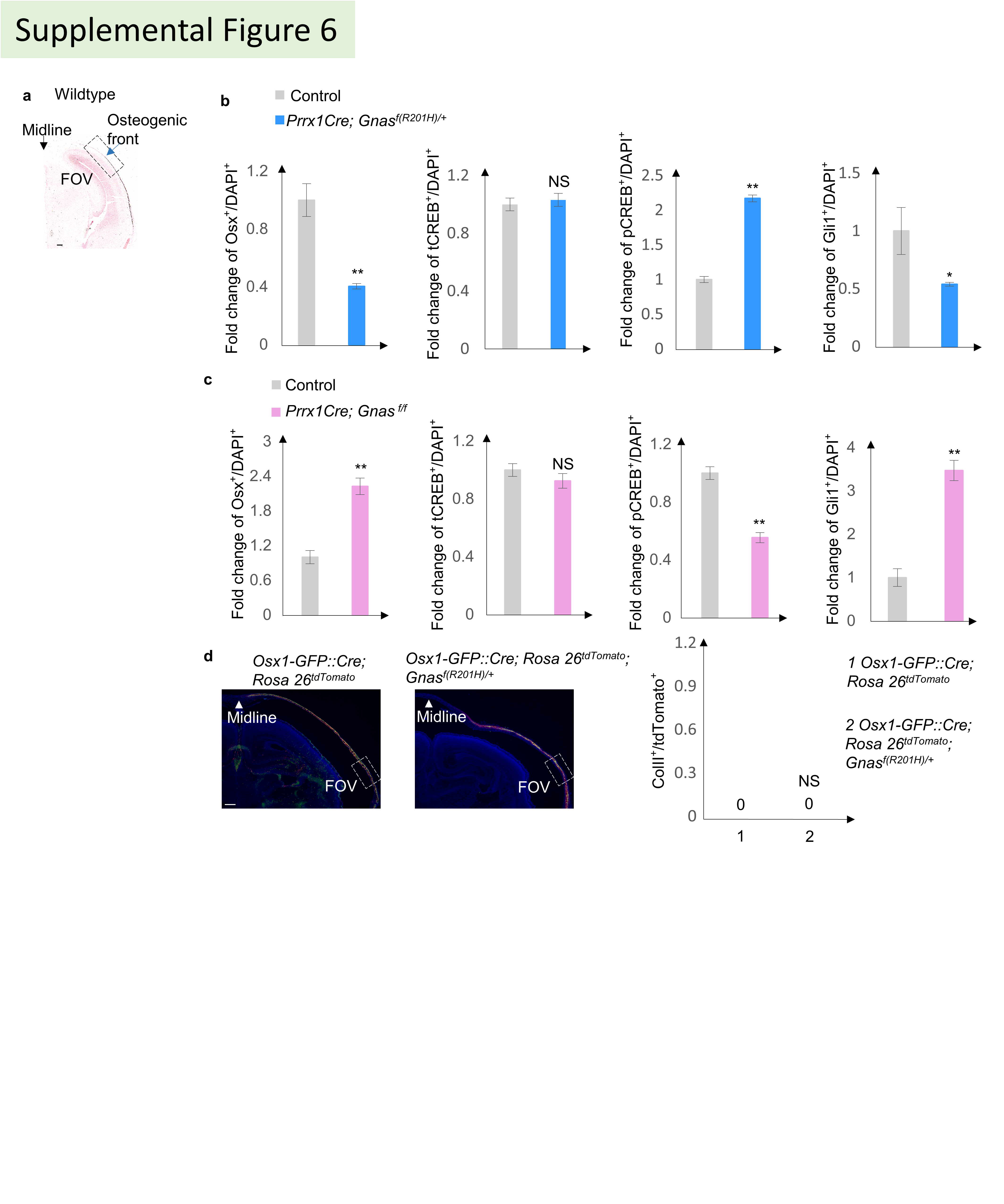

Supplement: Supplementary file 6 — Quantification of immunostaining at osteogenic front at E15.5 and P0 [file 41413_2018_34_MOESM6_ESM.tif]
